# Sertraline-Induced Mood and Behavioral Activation in Two Adults With Prader–Willi Syndrome

**DOI:** 10.1155/crps/9811985

**Published:** 2025-06-02

**Authors:** Janice Forster

**Affiliations:** Pittsburgh Partnership, Specialists in PWS, Pittsburgh, Pennsylvania, USA

**Keywords:** COVID-19, epigenetics, mood and behavioral activation, neuropsychiatric phenotype, pharmacogenomics, Prader–Willi syndrome genotype, sertraline, SSRIs

## Abstract

**Objective:** Risk for mood and behavioral activation (MBA) due to selective serotonin reuptake inhibitors (SSRIs) is multiply determined in persons with Prader–Willi syndrome (PWS) due to underlying epigenetic and pharmacogenomic factors that affect medication response. Further, age and molecular subtype of PWS are predisposing factors, as there is a >60% risk for bipolar disorder onset prior to age 30 among those with maternal uniparental disomy (mUPD). This article presents two cases of MBA due to sertraline prescribed to treat anxiety in these adults with PWS (mUPD).

**Methods:** Literature review, clinical experience, and data from group home behavior logs inform this case report. The assent of the patients and the consent of their parents (legal guardians) were obtained for this publication.

**Results:** In these two cases, the gradual onset of MBA occurred over 1 year as the dose of sertraline was increased causing irritability, sleep disturbance, increased intensity of hyperphagia, and other phenotypic behaviors. These clinical signs were attributed to the stress of COVID-19 shutdown that resulted in loss of community activities for work, socialization, leisure, and exercise. But after sertraline was discontinued, activation resolved. Mood-stabilizing medication was required for a return to baseline, as sertraline may have unmasked or exacerbated an underlying bipolar diathesis.

**Conclusion:** Sertraline and other SSRI medications can cause MBA in patients with PWS at typical starting doses, although risk for adverse effects increases with higher doses. Age is a contributing factor. Knowing the genetic subtype of PWS is essential for making clinical decisions about pharmacotherapy, and results of pharmacogenomic testing may inform the selection of medication, dose, and schedule of administration.

## 1. Background

Prader–Willi syndrome (PWS) is a rare (1:15,000 births) genetic and complex neurodevelopmental disorder with a unique neuropsychiatric phenotype that changes across development and varies with the molecular genetic subtype. PWS is caused by the absent expression of multiple contiguous genes from the paternal chromosome in the 15q11-q13 region because of deletion (DEL), maternal uniparental disomy (mUPD), or imprinting center defect (ICD) [1]. The loss of expression of these imprinted genes causes hypothalamic dysfunction that results in decreased arousal, impaired sleep/wake cycles, diminished hunger/satiety and thirst, temperature dysregulation, decreased sensitivity to pain and disgust, life-long stress sensitivity, and inability to adapt to environmental change. Infants are hypotonic at birth and display hypogonadism and growth hormone deficiency. They display failure to thrive that requires strict calorie management often by tube feedings. When treatment with growth hormone begins in infancy, it improves the age of acquisition of developmental milestones, cognitive development, body composition, and normalizes facial dysmorphisms, but it does not alter the trajectory of the behavioral phenotype [2, 3]. Despite feeding problems in infancy, children begin to show an interest in eating that results in hyperphagia by age 8 leading to obesity if food access is not secured in the environment [4].

The behavioral phenotype of PWS consists of food-related behaviors, excessive/repetitive behaviors, cognitive rigidity, impulsive/disruptive behavior, anxiety/mood symptoms, and skin picking, which constitute the PWS Personality [5–7]. Anxiety is a common feature throughout development [8]. Psychological food security (no doubt about what, how much, and when the child will eat and no chance or opportunity to get additional food) manages expectations around food, which decreases anxiety and food related behaviors [9]. Temper tantrums and repetitive behaviors increase during childhood, peak around age 10 years, and remain throughout adulthood [6, 8, 10]. During the late adolescent and young adult years, patients with the DEL molecular subtype are susceptible to depressive disorders, while those with mUPD are more likely to experience cyclic psychosis or bipolar mood disorders [11, 12]. In fact, among persons with PWS who have the mUPD molecular subtype, >60% have acquired a diagnosis of bipolar disorder by the age of 30 [13].

Selective serotonin reuptake inhibitors (SSRIs) have been used frequently to target excessive/repetitive behaviors, anxiety/mood symptoms, and impulsive/disruptive behavior (temper tantrums) in patients with PWS of all ages [6, 10, 14]. Adverse effects at typical doses are not uncommon among neurodevelopmental disorders, leading the informed clinician to start at lower than the typical starting dose and titrate slowly [15]. This medication sensitivity is multiply determined in patients with PWS. First, there is an epigenetic decrease in the function of the serotonin 2C receptor due to abnormal editing caused by the absence of expression of a snoRNA in the critical region on Ch 15 [16]. Further, the low activity of the inhibitory serotonin 2C receptor creates an imbalance with the excitatory serotonin 2A receptor in the prefrontal cortex, resulting in age-related mood activation [17]. In addition, there is a bias toward excitation versus inhibition in the anterior cingulate cortex because of a decrease in GABA receptors and GABA tone [18, 19]. Finally, pharmacogenomic testing in a case series of individuals with PWS found statistically significant differences in pharmacokinetic and pharmacodynamic gene polymorphisms when comparing the results from DEL and mUPD with the normative population [20, 21]. For example, there were more gene polymorphisms coding for the poor or intermediate metabolizer phenotype of some cytochromes, for example, CYP2D6, indicating greater medication sensitivity and increased risk for adverse effects.

The symptoms of activation syndrome associated with SSRI treatment are increased arousal, restlessness, impulsivity, disinhibition, anxiety, panic attacks, somatic complaints, irritable mood, emotional lability, and decreased sleep [22]. The time of onset of mood and behavioral activation (MBA) can be within 2 to 3 weeks of initiating or increasing the dose of SSRI, although symptoms can emerge at any time during treatment with a median of 3 months after exposure [23]. Symptoms of MBA are often confused with an exacerbation of target symptoms or the emergence of antidepressant triggered hypo/mania associated with bipolar disorder [24]. Among patients with anxiety or depression and possible bipolar diathesis, there is an increased risk for bipolar hypo/manic activation when SSRI or NSRI antidepressants are prescribed [25]. SSRI-induced bipolar disorder is most often associated with elation and grandiosity indicative of a hypo/manic mood shift, whereas MBA is associated predominantly with an irritable mood and psychomotor agitation [22]. In a case series of 100 consecutive admissions to an inpatient rehabilitation program for patients with PWS, MBA occurred in one-third of patients who were receiving any SSRI, NSRI, or aripiprazole regardless of age, sex, or genotype [26]. In fact, among those who activated, 25% received fluoxetine, 20% received sertraline, 18% received aripiprazole, and 14% received citalopram. The phenomenology of MBA in patients with PWS differs from cyclic psychosis or bipolar disorder by the *gradual onset* of an increased intensity of baseline phenotypic behaviors concurrent with the emergence of MBA symptoms. Cyclic psychosis is rapid in onset and recovery, presenting with the sudden onset of confusion, mood instability, paranoid delusions, motor symptoms, failure to eat, and loss of ability to perform grooming or dressing activities [27]. In bipolar hypo/manic disorder, the mood switch is rapid in onset with decreased sleep, increased goal directed behavior, grandiose delusions, and risky behaviors with high potential for harm; there is gradual improvement with appropriate treatment that may be followed by a mood shift to depression [28]. Management of MBA requires the discontinuation of the activating agent, and the time to recovery usually exceeds that predicted from the pharmacokinetics of the agent. Additional treatment with mood stabilizers or antipsychotics may be required. All these psychiatric disorders are exacerbated by stress, and patients with PWS have extreme stress sensitivity.

During the developmental years, centers of excellence for PWS provide multidisciplinary clinics for children and adolescents that include pediatric specialties of endocrinology (among others) and child and adolescent psychiatry to evaluate and manage excessive daytime sleepiness, attention problems, behavioral difficulties, and mood symptoms [29]. Adults with PWS age out of these multidisciplinary clinics, and given the high incidence of psychiatric comorbidities, patients are seen by local mental health providers who have limited knowledge and experience with the syndrome. The adverse events highlighted in this article are *the most common reason* for peer-to-peer consultations coordinated through PWSA-USA linking the author with prescribing clinicians.

The data presented in this case series were obtained from the psychiatric and behavioral history reported by the parents of both patients and from the daily behavior logs from the group home in the second case. Both patients were seen by other clinicians prior to referral to the author for evaluation and treatment. The case formulation and data synthesis were informed by clinical experience, a literature review (PubMed: Prader–Willi syndrome, sertraline, mood and behavioral activation, SSRI), and pharmacogenomic testing by GeneSight (AssureRx Health Inc.). The assent of the patients and the consent of their parents who are their legal guardians were obtained for this publication.

## 2. Results

These two cases illustrate the gradual onset of MBA occurring over 1 year (2022–2023) as the dose of sertraline was increased to treat anxiety. Early signs of MBA were attributed to life stress associated with COVID-19 shutdown that resulted in loss of community activities for work, socialization, leisure, and exercise. Then, there was an increase in the intensity of phenotypic behaviors (food seeking, cognitive rigidity, repetitive questioning, and tantrums) associated with PWS together with the onset of symptoms of MBA (irritability, emotional reactivity, impulsivity, hostility, anxiety with somatic complaints, hyperarousal, and sleep disturbance) with some gender differences. Later, symptoms of psychotic proportion with delusions were noted. After the sertraline dose was tapered and discontinued, symptoms of MBA decreased, but additional mood stabilizing medication was prescribed for a full return to baseline mood and behavior.

### 2.1. Case Report #1

A 32-year-old woman with PWS (mUPD) and mild intellectual disability resided at home with her parents. She had been receiving sertraline at a daily dose of 12.5 mg for treatment of anxiety for nearly 20 years with favorable response. During the COVID-19 pandemic, her stress increased due to the loss of her day program, decreased attendance at community activities, and multiple family losses. To target anxiety related to stress, sertraline dose was increased to 25 mg and then 37.5 mg over the course of 1 year. The patient began to experience anxiety attacks with increasing frequency when she left the home, consisting of sensory overstimulation with intolerance of previously enjoyable community activities, feeling unwell, and pleading with her parents to take her to the hospital for evaluation. Serial evaluations in the emergency department (ED) did not find a medical cause. Transporting her in the car was dangerous due to her tantrums and impulsivity, as she grabbed the gear shift to reverse direction, nearly precipitating an accident. At home, she experienced the new onset of sleep continuity disturbances with food seeking; increased repetitive questioning; intolerance of being alone; and attempts to control other's behavior about food, the daily schedule, and the manner of task completion. Her parents had exhausted all behavioral strategies that were effective previously.

On evaluation by this clinician, she was anxious, irritable, and tearfully labile but not depressed or psychotic. She was argumentative, impatient, and easily frustrated. There was passive suicidal ideation. She did not display tremors, dystonia, or flushing (although she complained that she always felt hot and serotonin syndrome was in the differential); vital signs were stable. She complained of back pain for which she was being seen by a chiropractor. Past medical history included spinal fusion for scoliosis. There was no family history of mood disorder or psychosis.

Differential diagnosis was personality change secondary to PWS (mUPD) ICD-10 CM F07; MBA due to a medication (sertraline) ICD-10 CM F19.9; and R/O bipolar mood disorder ICD-10 CM F34 [30].

Pharmacogenomic testing was obtained and was significant for the poor metabolizer phenotype of CYP2B6, the principle metabolic pathway for elimination of sertraline, and the intermediate activity phenotype of methylene tetrahydrofolate reductase, *MTHFR* polymorphism (C677T, C/T), which results in decreased conversion of dietary folic acid to L-methyl folate, a precursor for neurotransmitter synthesis, and cofactor for energy metabolism in the brain, predisposing to mood disorder (major depression >> bipolar disorder) and psychosis [31]. Pharmacodynamic genes of serotonin 2A receptor *HRT2A* and the alpha-2-adrenergic receptor *ADRA2A* had polymorphisms with reduced sensitivity and response. Catechol-o-methyltransferase gene *COMT* (VAL/VAL polymorphism) has been associated with risk for generalized anxiety disorder in females [32, 33]. The serotonin transporter gene *SLC6A4* had the long promoter polymorphism L/L associated with an enhanced response to SSRIs, especially in females [34].

To address MBA, the dose of sertraline was tapered and discontinued. Behavior improved over the ensuing weeks with decreased frequency of impulsive behavior, tantrums, hyperarousal, and somatic complaints. Anxiety continued to interfere with daily function with repetitive questioning and demands. Lorazepam up to 1 mg TID was ineffective (*UGT2B15* activity was normal extensive metabolism phenotype). Low doses of olanzapine (2.5 mg/day) caused akathisia (*CYP1A2* activity was normal extensive metabolism phenotype) and was discontinued. Gabapentin was highly effective for managing anxiety and residual sleep disturbance. However, the patient continued to display a high level of goal-directed behavior and bossiness (need for control and need to assert dominance) that was eventually recognized as a manifestation of grandiosity. Lithium carbonate was initiated and titrated to a therapeutic level with significant improvement in mood and return to baseline behavior over a recovery interval of several months. L-Methyl folate 7.5 mg daily was prescribed also to improve mood. Environmental management focused on increasing the level of predictability and certainty with the daily schedule, especially around food and other rewarding activities.

After 1 year of follow-up, the patient continues to do well and has returned to her previous level of community integration. She is receiving therapeutic doses of lithium, gabapentin, and L-methyl folate. She is sleeping well, and her weight is back to baseline. Her parents' level of stress has normalized.

### 2.2. Case Report #2

A 31.6-year-old man with PWS (mUPD) and mild intellectual disability was the only resident of a specialized group home for persons with PWS; his behavior was stable for years. At his baseline, he was having one episode of physical aggression per month and one episode of verbal aggression per week. He had been receiving lithium ER 750 mg daily and valproic acid 625 mg twice daily for treatment of impulsive aggression and mood elevation manifested by increased goal directed behavior with elation. Clonazepam 0.25 mg in AM and 0.5 mg at HS and clonidine 0.1 mg at dinner time were prescribed for anxiety, and modafinil 100 mg in AM was prescribed for daytime sleepiness. After a series of stressors and COVID-19 restrictions led to community isolation and job loss, there was a gradual increase in anxiety with somatic preoccupation, and a new psychiatrist was assigned. Due to the patient's complaint of urinary frequency, the dose of lithium ER was decreased to 600 mg daily. Sertraline was started at a dose of 25 mg/day to target anxiety, and the dose of clonazepam was increased to 0.5 mg twice daily. The dose of valproic acid was maintained at 625 mg twice daily. The sertraline dose was increased every other month to a dose of 200 mg/day. Concurrently, the patient displayed an escalating frequency of disruptive behaviors with incidents of vulgar name calling; tantrums with slamming and pounding surfaces, kicking doors, and screaming for hours; verbal threats that staff should die; and predatory aggression with hitting and punching staff. One staff member was hospitalized. Transporting the patient in a car was dangerous as he grabbed the steering wheel and threw objects at the driver. Antecedents to outbursts were uncertainty about food choices, changes in daily schedule or staffing patterns, and unmet demands. He displayed early morning awakening. When the pattern of MBA was realized, the dose of sertraline was decreased precipitously to 100 mg/day, and risperidone 0.5 mg twice daily was started; the dose of lithium, clonazepam, and valproate remained the same. After 3 weeks, sertraline was discontinued, and the dose of risperidone was increased to 0.5 mg in AM and 1.0 mg at HS. Unfortunately, the mood and behavior disturbance persisted.

Days prior to the evaluation by this clinician, the patient was taken to a local ED for a “medication adjustment,” and the dose of risperidone was increased to 1 mg twice daily. Days later when this clinician evaluated the patient, he displayed extrapyramidal symptoms (EPS) of a shuffling gait, arms down by his side without an associated arm swing, mild dystonia, and cogwheeling of his upper extremities and slowed but symmetrical rapid alternating movements. Facial expression was limited by EPS. There were no buccal–lingual dyskinesias. His mental status exam was abnormal with slow processing speed, working memory deficit, and impulsive responding with decreased response inhibition. There was an increased rate of speech, but dysarthria and dysfluency limited production. Affect was constricted. His mood was dysthymic with sadness, irritability, uncertainty, somatic worries, and guilty preoccupation. He denied suicidality and homicidal ideation or plan. He denied hallucinatory experiences, but there were delusions with paranoia (persecution), grandiosity (he believed his voice influenced the action of others), and hyper-religiosity (he had recently changed his religious affiliation). His beliefs were fixed and egocentric.

Differential diagnosis was personality change secondary to PWS (mUPD) ICD-10 CM F07; MBA due to a medication (sertraline) ICD-10 CM F19.9; intermittent explosive disorder ICD-10 CM F63.81; and bipolar mood disorder ICD-10 CM F34 [30].

Although sertraline was discontinued, the patient was still symptomatic and receiving polypharmacy. Clinically meaningful drug–drug interactions were suspected to have occurred between clonidine, sertraline, and risperidone due to competitive binding at CYP2D6; sertraline and valproate due to competitive binding and modafinil inhibition of CYP2C9; sertraline increase due to modafinil inhibition of CYP2C19; sertraline decrease due to modafinil induction of CYP2B6; and altered efficacy of risperidone, modafinil, sertraline, and clonazepam due to competitive binding at CYP3A4 [21]. Pharmacogenomic testing was obtained and identified the *CYP2D6* polymorphism associated with the intermediate metabolizer phenotype resulting in decreased elimination of sertraline, risperidone, and clonidine [35]. Pharmacodynamic gene polymorphisms were identified for the serotonin transporter promoter, *SCL6A4* (s/s), which confers increased stress sensitivity and altered effects of SSRIs; the serotonin 2A receptor *HTR2A* (-1438G >A, G/G) that confers increased risk for adverse effects of SSRIs, hypertension, and metabolic syndrome; the alpha-2-adrenergic receptor *ADRA2A* (-1291G >C, C/C), resulting in moderately reduced response to medications active at this receptor (e.g., clonidine) [36]; and the methylenetetrahydrofolate reductase *MTHFR* polymorphism (C677T, C/T) resulting in intermediate activity with risk for mood disorder (major depression >> bipolar mood disorder) and psychosis [31]. The result for the catechol-o-methyltransferase gene *COMT* revealed the VAL/MET polymorphism, which is typical for the normative population.

**Figure 1:**
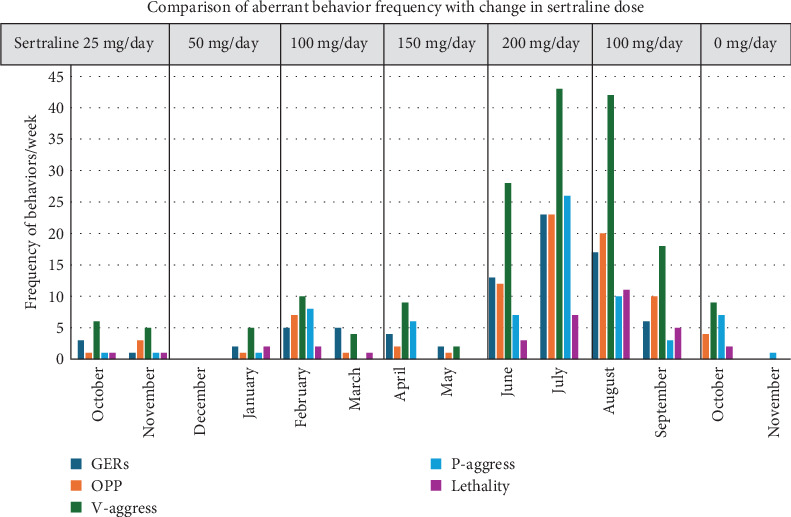
Comparison of the frequency of aberrant behaviors with the change in sertraline dose over time. The disruptive behaviors described in the narrative record of care at the group home have been itemized and tallied to establish a frequency for each week across 1 year's time (no data for December). General event reports (GERs) are critical incident reports that imply a degree of severity; OPP are oppositional behaviors with arguing, fault finding, asserting dominance, and appealing to authority figures (calling staff supervisor or parent); V-aggress is verbal aggression with screaming including name calling, use of profanity, use of racial slurs, and vulgarity; P-aggress is disruptive behavior escalating to property destruction (kicking doors and punching walls) and physical aggression; and lethality is defined by threats of retaliation, homicidal wishes or threats, and dangerous behaviors with risk of injury to self and others.

Laboratory testing revealed a trough serum lithium level of 0.5 µm/L (low). Initial management by this clinician was to titrate the dose of lithium ER to a serum level of 1.0 µm/L, which was achieved at a dose of 900 mg in the morning and 600 mg at dinnertime daily. L-Methyl folate was prescribed. The patient continued to receive the same doses of valproic acid and clonidine. His behavior improved gradually over several months, and doses of risperidone and clonazepam were decreased. Modafanil was discontinued. Greater consistency in the execution of the daily plan was emphasized. Sensory motor stimulation and relaxation exercises were prescribed.  Figure 1 demonstrates the patient's clinical course. As the dose of sertraline was increased, aberrant behaviors escalated and then diminished after sertraline dose was reduced and finally discontinued.

After 1 year of follow-up, the patient has continued to improve, and his mood and behavior are stable without physical or verbal aggression; oppositional behaviors are at low frequency. He is receiving therapeutic doses of lithium; sodium valproate has been decreased to 500 mg BID; risperidone was reduced to 0.25 mg per day, and clonidine dose is 0.05 mg TID. Clonazepam was tapered and discontinued. He continues to receive omega-3-fish oils and L-methyl folate. He is awaiting a supported job placement in the community. He enjoys socialization experiences with friends who have PWS and attends local sporting events with staff.

## 3. Discussion

This case report describes two adult patients with PWS (mUPD) who developed MBA when sertraline was prescribed to treat anxiety. The first patient (Case #1) received low doses of sertraline prescribed by the primary care provider, and the second patient (Case #2) received increasing doses of sertraline in addition to other medications prescribed by a psychiatrist consulting to the group home. Stress was the antecedent event, precipitated and perpetuated by the environmental lockdown associated with the COVID-19 pandemic that interfered with the predictability and consistency of the supportive and experienced caregiving environments. In these two cases, the onset of MBA was gradual over 1 year's duration and related to the dose titration of sertraline. Unfortunately, the treating clinicians missed the emerging symptoms of MBA. First, there was an increased intensity of phenotypic behaviors (stress sensitivity, emotional reactivity, cognitive rigidity, tantrums, and food-related symptoms). Then sleep disturbance emerged with discontinuity (female) and early morning awakening (male). In the female patient, anxiety symptoms became extreme with somatization, panic attacks, egocentrism, and need for control. In the male patient, asserting dominance with arguments, disruptive behavior, and aggression predominated. Behavioral data showed a clear escalation following dose increments of sertraline to the point of paranoid delusions that required additional pharmacotherapy (risperidone). In both cases, the predominant mood state was irritable. It was not until sertraline was tapered and discontinued that behavior improved, although mood-stabilizing medication titrated to a therapeutic serum level was required for a return to baseline. The time interval to improvement was much greater than predicted by the pharmacokinetics of sertraline, as the time interval required for symptom remission after MBA is often the same as the time interval leading to activation [37]. Pharmacogenomic factors of low activity and slow metabolizing polymorphisms as well as drug–drug interactions were factors predisposing to the poor elimination of sertraline resulting in the adverse effect of MBA.

In addition to causing MBA, sertraline appears to have unmasked a bipolar diathesis in the first patient, although she was already 32. The second patient was diagnosed with bipolar disorder after a past episode of hypo/mania and was at high risk for a mood switch with sertraline. Neither patient had a family history of bipolar disorder. Both patients had the mUPD molecular subtype of PWS that carries age-related risk for bipolar disorder. The phenomenology of MBA in these two cases is differentiated from an episode of bipolar mania by the predominance of irritable mood and hyperarousal that diminished when sertraline was tapered and discontinued.

These cases were selected because of their complexity and because they exemplify issues related to the starting dose and titration of medication. Low doses of SSRI medications are commonly prescribed using the mantra of *start low*, *go slow* to augment the behavioral and ecoenvironmental interventions that manage the cognitive, mood, and behavioral symptoms associated with PWS phenotype [6, 10, 14]. The patient in Case #1 is consistent with this practice in that she was prescribed low dose sertraline (12.5 mg) with good response for 20 years. In a retrospective chart review of MBA in a case series of patients with PWS age 11–27, activation occurred at greater than typical starting doses of SSRIs, for example, 25 mg of sertraline [38]. Further, Deest and colleagues reported a clinical case series of adults with PWS (mostly DEL) where the addition of low dose sertraline (25 mg in most cases) resulted in behavioral improvement over the ensuing 6 months and allowed for a subsequent reduction in the doses of concurrent antipsychotic and mood-stabilizing medications [39]. Sertraline has some minimal effect on norepinephrine and greater effect on dopamine activity than other SSRIs, so adding it to ongoing drug regimens may potentiate positive effects on mood and behavior [40]. However, polypharmacy rarely improves symptom management due to the potential for drug–drug interactions [41].

Unfortunately, the use of multiple classes of psychotropic medication is commonplace in patients with PWS due to off-label use (without licensed indication) and psychiatric comorbidity [6, 10]. The Paving the Way for Advancements in Treatment and Health (PATH) for PWS study was initiated in 2015 by the Foundation for Prader–Willi Research to study adverse medical events and psychotropic medication use [14]. There were 647 enrollees across multiple age groups yielding the following data: in the 5–11 year group (*n* = 218), 33.6% were using psychotropic medications (13% antidepressants); in the 12–18 year group (*n* = 177), 49.1% were using psychotropics (33.6% antidepressants); and among 252 patients older than 18 years, 65.5% were using multiple psychotropic medications (48% antidepressants). Polypharmacy increased with age and included antidepressants (mostly SSRIs), antipsychotics, mood stabilizers, antianxiety agents, and sedative/hypnotics. As previously mentioned, drug–drug interactions are more likely to occur in patients with PWS due to epigenetic and pharmacokinetic differences compared to the normative population [21]. Also, polypharmacy contributes to decreased heart rate variability that increases morbidity and mortality [42].

Finally, the primary stressor in both cases was the impact of the COVID-19-related shutdown that resulted in change of routine, loss of consistency and predictability of staff, and decreased availability of community activities for work, socialization, leisure, and exercise. The pandemic had a profound, deleterious effect on care systems and community providers serving individuals with intellectual disabilities [43]. Staff shortages were exacerbated due to illness, burnout, and attrition [44, 45]. Retrospective studies on the psychological impact of lockdown identified an increase in mental health and behavior problems in more than half of adults with PWS living in their parents' homes [46, 47]. Behavior problems persisted in approximately one-third after lockdown was over [46]. Further, in a cohort of 89 respondents, Wieting et al. [47] identified irritability in 55%, sadness in 43.8%, and anxiety in 38.2%. Another study noted more positive effects on health and well-being with a lesser increase in behavior disorders among persons with PWS [48]. Unfortunately, there were no comparable studies in the United States. A study from the United Kingdom noted an overall increase in prescribing rates for all classes of psychotropic medications among individuals with intellectual disability during COVID-19 lockdown [49]. There was a cautionary note that the stress of COVID-19 may have resulted in an over-reliance on medication rather than consultation with multidisciplinary teams for alternative interventions. Fortunately for the patient in Case#2, it was the consultation with the behavioral team that lead to the meticulous data collection showing exacerbation of mood and behavior, eventually resulting in the taper and discontinuation of sertraline. The improvement in behavior was concurent with the discontinuation of sertraline, eventhough the stress of COVID-19 lockdown was ongoing.

## 4. Limitations

Although pharmacogenomic testing on these patients provided some understanding of pharmacokinetic factors affecting the metabolism of sertraline, serum levels were not obtained. Studies suggest that the increase in serum drug levels of sertraline is linear across dose increments, and the change in serum level is predicted by the phenotypic activity of cytochrome polymorphisms [50–54]. Therapeutic drug monitoring of SSRI medications is not as yet the standard of care in clinical practice, but some psychiatrists are advocating for its use [55].

The stress of COVID-19 lockdown cannot be minimized in these cases. There was a reflexive approach to use medication to manage stress. Although anxiety was the target symptom, neither of these patients responded to anxiolytics. There were other recommendations to re-establish the consistency and predictability of the daily routine, especially around exercise, food, and other rewarding activities. But the stress on the parents and group home staff was so extreme, they were limited in their capacity to make the necessary changes.

## 5. Conclusion

This report of MBA in two adults with PWS demonstrates the risk of adverse effects associated with the use of sertraline at different doses in persons with PWS who have predisposing factors of age, mUPD molecular subtype, bipolar comorbidity, and at risk pharmacogenomic phenotypes with potential for drug–drug interactions. The adverse effect of MBA is more likely to occur when prescribing clinicians are unfamiliar with the unique neurochemistry affecting the serotonin system in PWS, and they fail to recognize the gradual pattern of increasing intensity of phenotypic behaviors as the dose of medication is titrated. Prompt recognition of MBA symptoms and the timely discontinuation of the activating agent decreases the duration of the activation episode and minimizes risk for bipolar activation in susceptible patients. Our collective caregiving experience during COVID-19 has empahsized an over-reliance on medications to compensate for gaps of knowledge about alternative interventions, environmental management strategies, or a dearth of providers with this specialized acumen [49].

## Data Availability

The data that support the findings of this study are available on request from the corresponding author. The data are not publicly available due to privacy or ethical restrictions.
